# Analysis of the immune-related transcriptome of a lophotrochozoan model, the marine annelid *Platynereis dumerilii*

**DOI:** 10.1186/1742-9994-4-18

**Published:** 2007-07-06

**Authors:** Boran Altincicek, Andreas Vilcinskas

**Affiliations:** 1 Institute of Phytopathology and Applied Zoology, Interdisciplinary Research Center, Justus-Liebig-University of Giessen, Heinrich-Buff-Ring 26-32, D-35392 Giessen, Germany

## Abstract

**Background:**

The marine annelid *Platynereis dumerilii *(Polychaeta, Nereididae) has been recognized as a slow-evolving lophotrochozoan that attracts increasing attention as a valuable model for evolutionary and developmental research. Here, we analyzed its immune-related transcriptome. For targeted identification of immune-induced genes we injected bacterial lipopolysaccharide, a commonly used elicitor of innate immune responses, and applied the suppression subtractive hybridization technique that selectively amplifies cDNAs of differentially expressed genes.

**Results:**

Sequence analysis of 288 cDNAs revealed induced expression of numerous genes whose potential homologues from other animals mediate recognition of infection (e.g. complement receptor CD35), signaling (e.g. myc and SOCS), or act as effector molecules like ferritins and the bactericidal permeability-increasing protein. Interestingly, phylogenetic analyses implicate that immune-related genes identified in *P. dumerilii *are more related to counterparts from Deuterostomia than are those from Ecdysozoa, similarly as recently described for opsin and intron-rich genes.

**Conclusion:**

Obtained results may allow for a better understanding of *Platynereis *immunity and support the view that *P. dumerilii *represents a suitable model for analyzing immune responses of Lophotrochozoa.

## Background

Comparative immunology has become an integrative discipline in zoology and has gained wide acceptance in biological sciences [1]. Research on molecular aspects of innate immunity was mainly performed on Ecdysozoa (*Drosophila *[2] and *Caenorhabditis *[3]) or Deuterostomia (fish and murine models), whereas information about immune responses from Lophotrochozoa is rather fragmentary and needs further elucidation. Identification of immune-related genes from lophotrochozoan species was performed by differential display RT-PCR in the leech *Theromyzon tessulatum *(Annelida, Hirudinea) [4] and by subtractive suppression hybridization, the same method as used in this study, in the oysters *Crassostrea virginica *and *C. gigas *(Mollusca, Bivalvia) [5] and the snail *Biomphalaria glabrata *(Mollusca, Gastropoda) [6,7]. Here, we used the marine polychaete *Platynereis dumerilii *for targeted screening of the immune-related genes because this annelid has been emerged as an ideal model organism for evolutionary analyses of a slow-evolving Bilateria [8,9].

Since genomic data become available from an increasing number of invertebrates such as the sea urchin *Strongylocentrotus purpuratus *[10], comparative immunology attracted renewed attention. But screening for immune-related genes in genomic or EST databases has one major disadvantage. It allows only the identification of genes that share sequence similarities with known immune-related proteins from other organisms. Here, we used an experimental approach for targeted screening of immune-related genes in *P. dumerlii *that allows selective amplification of genes with enhanced expression rates in response to septic injury. For this purpose, we injected crude bacterial lipopolysaccharide (LPS), a potent and widely used elicitor of innate immune responses, into the coelom of adult *Platynereis*, isolated their RNA 8 hours post challenge, and analyzed immune-related transcripts by a subtractive suppression hybridization approach. This PCR-based method selectively amplifies cDNAs of differentially expressed genes and simultaneously suppresses amplification of common cDNAs. This technique has been proven as a suitable tool for identification of immune-related genes in a variety of animals including the apterygote insect *Thermobia domestica *[11], the mosquito *Anopheles gambiae *[12], the tsetse fly *Glossina morsitans *[13], the lepidopterans *Galleria mellonella *and *Manduca sexta *[14,15], the oysters *C. virginica *and *C. gigas *[5], the snail *B. glabrata *[6,7], and the zebrafish *Danio rerio *[16]. Here, we describe the identification of immune-related genes from *P. dumerlii *which may provide novel insights into polychaete innate immunity.

## Results and Discussion

### Subtracted cDNA library of immune challenged *P. dumerilii*

**Figure 1:**
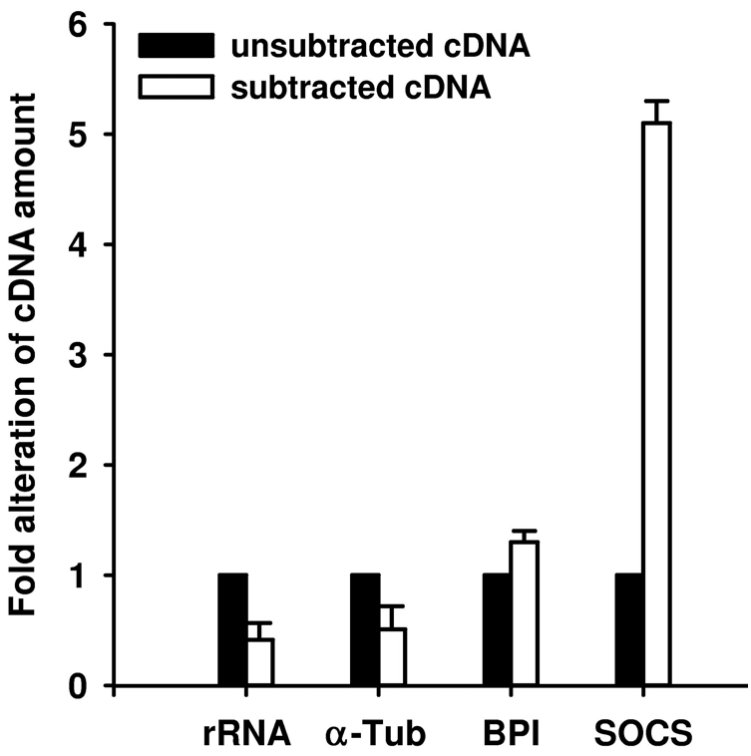
**Confirmation of the enrichment of immune-related transcripts in the subtracted cDNA library by quantitative real-time PCR analysis**. The relative amount of cDNAs of 18 S rRNA, α-tubulin, BPI, and SOCS in the subtracted cDNA library (white bars) is shown relative to their amount in unsubtracted cDNA library (black bars). The cDNA amount of the house-keeping genes 18 S rRNA and α-tubulin were reduced about 2.5 fold by the subtraction procedure. In contrast, potentially immunity-related genes BPI and SOCS were found to be 1.3 and 5.1 fold enriched in the subtracted cDNA library, respectively. Results represent mean values of three independent determinations ± S.D.

A subtracted cDNA library enriched in immune-inducible genes was constructed using purified RNA from eight adult *P. dumerilii *injected with bacterial LPS and eight untreated animals. To induce strong and broad immune responses we injected a commercially available purified LPS preparation that is known to contain impurities like nucleic acids, proteins, and peptidoglycans and that is commonly used as elicitor in vertebrate and invertebrate research. In order to confirm that the subtraction process has been performed efficiently, we analyzed the abundance of transcripts of the house-keeping genes 18 S rRNA and α-tubulin and for two genes, the bactericidal permeability-increasing protein (BPI) and suppressor of cytokine signaling (SOCS). The latter ones were found to be induced in response to immune challenge in *Platynereis*. Quantitative real-time PCR analyses revealed that transcripts of 18 S rRNA and α-tubulin were reduced 2 to 3 fold, whereas transcripts of BPI and SOCS were enriched for 1.3 and 5.1 fold, respectively (Fig. 1). This is in agreement with values from the protocols of the manufacturer and indicates successful subtraction of the cDNA library. A total of 288 clones from this library were randomly picked and subjected to colony PCR. Plasmids of 140 colonies that were positively screened in blot hybridization indicating immune-induced expression of corresponding genes were isolated and sequenced. Obtained sequences were deposited at EMBL-European Bioinformatics Institute, compared to databases of the National Center for Biotechnology Information using BLASTX program, and summarized in table 1. InterProScan at the EMBL-European Bioinformatics Institute was used for an integrated search in PROSITE, Pfam, and PRINTS databases to predict conserved motifs, signal sequences, and transmembrane regions.

**Table 1:** cDNAs from the subtracted *P. dumerilii *library

Cluster	EMBL accession #	Highest BlastX match	*E *value	PFAM/InterPro	PEPD EST clone ID
*Defense proteins*					
	AM697673	P17213: Bactericidal permeability-increasing protein (*Homo sapiens*)	0.036	BPI2	
	AM697674	P42577: Soma ferritin (*Lymnaea stagnalis*)	6e-69	Ferritin	AAAY-59-09-D
	AM697675	P08267: Ferritin heavy chain (*Gallus gallus*)	2e-33	Ferritin	AAAY-70-04-A
	AM697676	P80060: Protease inhibitors (*Locusta migratoria*)	0.0017		AAAC-30-10-H
	AM697677	Q5MGQ0: Defense protein 1 (*Lonomia obliqua*)	5e-05	Reeler	
	AM697678	P91778: Alpha-amylase (*Pecten maximus*)	2e-48	Alpha-amylase	
*Signal transduction*					
	AM697681	P17927: Complement receptor type 1 (CD35, *Homo sapiens*)	2e-06	Sushi	
	AM697682	P51675: C-C chemokine receptor type 1 (CD191, *Mus musculus*)	2e-07	7tm_1	
	AM697683	Q7TPQ9: Arrestin domain-containing protein 3 (*Mus musculus*)	2e-22	Arrestin_C	
	AM697684	Q9PW70: Suppressor of cytokine signaling (*Gallus gallus*)	3e-12	SH2	
	AM697685	Q80T79: CUB and sushi domain-containing protein 3 (*Mus musculus*)	0.3		
	AM697687	P49709: Myc protein (*Carassius auratus*)	3e-19	Myc_N	
	AM697688	Q9DB43: Zinc finger protein-like 1 (*Mus musculus*)	2e-36	Zinc finger, RING-type	
	AM697690	Q08043: Alpha-actinin-3 (*Homo sapiens*)	1e-132	CH	AAAY-59-05-E
	AM697691	Q9PU47: Alpha-actinin-associated LIM protein (*Bos taurus*)	7e-07	PDZ	
	AM697692	Q3SYZ8: Alpha-actinin-associated LIM protein (*Gallus gallus*)	2e-05	PDZ	
*Apoptosis/Signaling*					
	AM697693	Q98943: Caspase-2 precursor (*Gallus gallus*)	6e-08	CARD	
*Protein and DNA biosynthesis*					
	AM697695	AAH76954: Polymerase (DNA directed), alpha 2 (*Xenopus tropicalis*)	1e-39	DNA_pol_E_B	AAAY-62-10-B
	AM697696	P07201: Ribonucleoside-diphosphate reductase small chain (*Spisula solidissima*)	4e-40	Ribonuc_red_sm	AAAY-66-21-F
	AM697697	AAV34860: ribosomal protein S4 (*Bombyx mori*)	6e-19	RS4NT	48-1-06-D
	AM697698	P69091: 60S ribosomal protein L18 (*Oreochromis niloticus*)	2e-56	Ribosomal_L18e	
	AM697699	P23403: 40S ribosomal protein S20 (*Xenopus laevis*)	6e-52	Ribosomal_S10	AAAY-68-04-F
*Respiratory-chain*					
	AM697700	Q8HXQ9: NADH-ubiquinone oxidoreductase 51 kDa subunit, mitochondrial (*Macaca fascicularis*)	1e-60	Complex1_51K	AAAC-41-02-C
*Membrane translocation*					
	AM697701	P25083: mitochondrial ADP/ATP translocator (*Solanum tuberosum*)	8e-63	Mito_carr	AAAD-41-05-D
	AM697702	P98198: phospholipid-transporting ATPase (*Homo sapiens*)	4e-59	ATPase, E1-E2 type	
	AM697703	Q6B865: ATP synthase c-subunit (*Ixodes pacificus*)	2e-37	ATP-synt_C	AAAY-48-20-H
	AM697704	Q9UN76: Amino acid transporter ATB0+ (*Homo sapiens*)	1e-33	SNF	
*Endosomal sorting*					
	AM697705	Q6NWF4: Vacuolar protein sorting protein 25 (*Danio rerio*)	0.61		
*Unknown functions*					
	AM697706	Q4PAT4: Hypothetical protein (*Ustilago maydis*)	1e-16		
	AM697707	P53858: Protein BNI4 (*Saccharomyces cerevisiae*)	0.1		
	AM697686	Hypothetical protein	NSM^1^		
	AM697689	Hypothetical protein	NSM		
	AM697694	Hypothetical protein	NSM		
	AM697679	Cationic α-helical putative antimicrobial peptide	NSM		
	AM697680	Disulfide bridged α-helical putative antimicrobial peptide	NSM		

^1^NSM, no significant match

Here, we describe the identification of *P. dumerilii *proteins that are potentially involved in recognition of infection, immune-related signaling, and effector mechanisms based on sequence similarities with known proteins from other organisms.

### Recognition of infection

**Figure 2:**
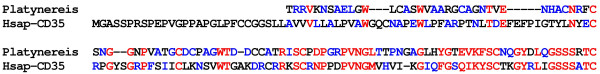
**Sequence alignment of potential *Platynereis *CD35 with human CD35**. The partial sequence of the putative CD35 from *Platynereis *(AM697681), was aligned with the amino-terminal sequence from human CD35 (Hsap-CD35, NP_000642). Red color indicates 80% consensus and blue color 50% consensus.

The complement system is an important component of innate immunity in vertebrates [17]. Because no complement genes were found in *Drosophila *and *Caenorhabditis *it has previously been suggested that the complement system is solely established in deuterostomes [18]. However, recently C3 was identified in the horseshoe crab (Arthropoda) and in corals (Cnidaria) implicating a much more ancient origin [18]. In this study we identified *Platynereis *transcripts encoding a protein that shares sequence similarities with human complement receptor type 1 (C3b/C4b receptor, CD35 antigen) (Fig. 2). In addition, we found cDNAs encoding a protein sharing highest sequence similarities with mouse C-C chemokine receptor type 1 (Macrophage inflammatory protein 1-alpha receptor, CD191 antigen) which is a G-protein coupled receptor for C-C type chemokines [19]. However, the similarities of obtained sequences to the potential counterparts are to short and to prevalent in similar receptors to construct a useful phylogenetic analysis; therefore, the given assignments should be taken with care.

### Signaling

The engagement of immune-related signaling pathways result in the massive production of cytokines and effector molecules that are necessary for pathogen elimination but that can also cause self-damage. Consequently, the regulation of exuberant immune responses is of great importance for the organism. In agreement, we found numerous genes in *P. dumerilii *that encode homologues known as negative regulators of immune responses. We identified the induced expression of a protein with highest sequence similarity to SOCS. SOCS proteins are negative feedback loop regulators of the evolutionary well-conserved JAK/STAT (Janus Kinase/Signal Transduction and Activator of Transcription) pathway that is required for cellular proliferation, stem cell maintenance and immune responses in both vertebrates and invertebrates [20,21]. In addition, it was shown for human SOCS-1 that it is involved in negative regulation of Toll-like receptor signaling [22].

Some among the identified sequences encode *Platynereis *proteins that contain an arrestin domain or share sequence similarities to alpha-actinin-3 and alpha-actinin-associated proteins, respectively. Arrestins were originally characterized as structural adaptor proteins that modulate the desensitization and trafficking of seven-membrane-spanning G protein coupled receptors and that have also been implicated in endocytosis of receptors and in crosstalk with other signaling pathways [23]. Alpha-actinin is a F-actin cross-linking protein that anchors actin to a variety of intracellular structures and is present in focal adhesion complexes [24]. Through these complexes integrins are able to transduce bi-directional signals between the extracellular matrix and intracellular signaling pathways essentially involved in cell shape change and motility of e.g. immune cells.

**Figure 3:**
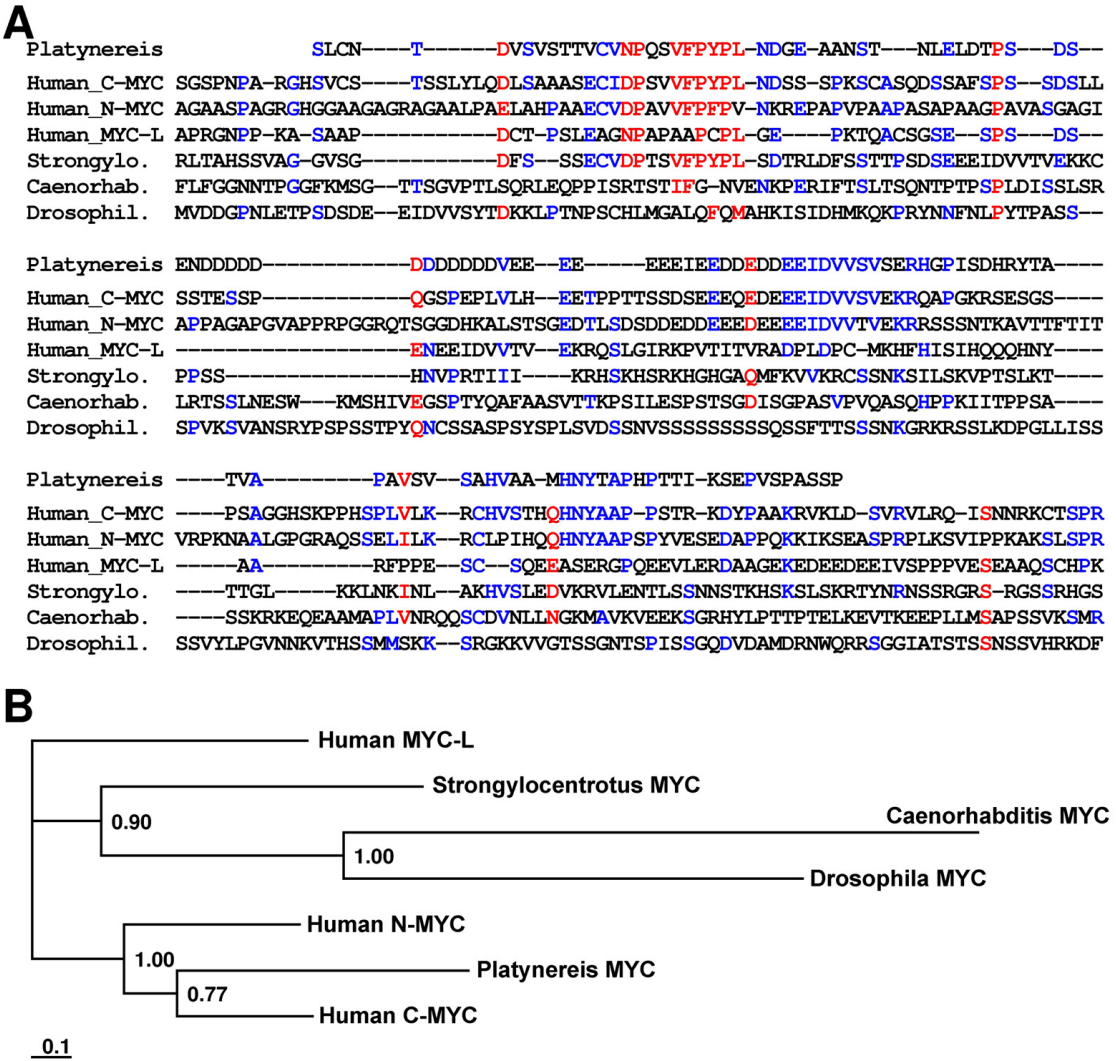
**Alignment and phylogenetic analysis of the myc oncogene**. (A) The partial protein sequence of myc from *P. dumerilii *(AM697687) was aligned with corresponding sequences from *S. purpuratus *(NP_999744), *D. melanogaster *(Q9W4S7), *C. elegans *(NP_001022773), human c-myc (P01106), and human n-myc (1202343A). The human myc-like protein (NP_001028253) was used as out-group. Red color indicates 70% consensus and blue color 40% consensus. (B) A Bayesian protein tree generated using the aligned protein sequences revealed that *Platynereis *myc shows highest relation to human c-myc. Posterior probabilities are plotted at the nodes. The scale bar represents the substitutions per site according to the model of amino acid evolution applied.

Interestingly, we further determined the induced expression of a protein with sequence similarities to the proto-oncogene myc (Fig. 3A) which is an important regulator of apoptosis in mammals. Phylogenetic analysis revealed that the *Platynereis *myc exhibits a higher relation to the human c-myc than to other analyzed myc homologues (Fig. 3B). The determined c-myc expression in activated human macrophages [25] reflects the need of a finely tuned balance between cell proliferation, cell division arrest, and apoptosis in the vertebrate immune cells [26]. Consistently with the importance of apoptosis regulation, we found immune-induction of potential homologues of caspase-2 and of the adenine nucleotide (ADP/ATP) translocator: (i) Caspase-2 is a member of a family of evolutionarily conserved cysteinyl proteases that mediate both apoptosis and inflammation through aspartate-specific cleavage of cellular substrates [27,28]; (ii) (ADP/ATP) translocator is an element of the mitochondrial permeability transition pore. This protein complex induces a sudden increase in permeability of the mitochondrial membrane resulting in cytochrome C release which is a key element in cell death [29].

### Effector molecules

We identified several transcripts of factors essentially involved in cell proliferation and in protein biosynthesis including the alpha-DNA polymerase, ribonucleoside reductase and several ribosomal proteins. This is plausible since production of effector molecules depends on the activation of cellular proliferation and translation and is commonly found in comparative analysis of innate immune responses [4-7,11,12,15].

**Figure 4:**
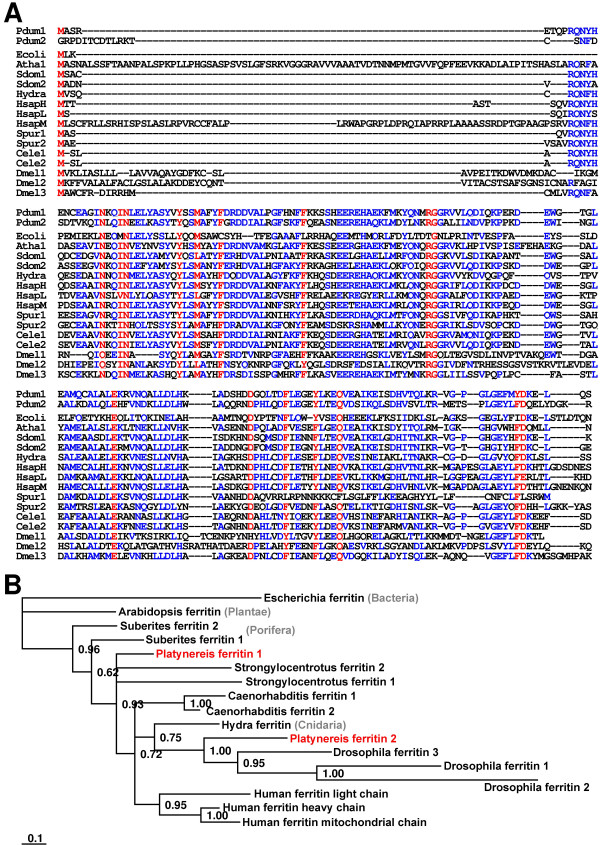
**Alignment and phylogenetic analysis of the ferritin isoforms**. (A) The protein sequences of the identified ferritin subunits from *P. dumerilii *(Pdum-1, AM697674; Pdum-2, AM697675) were aligned with corresponding sequences from *H. sapiens *(Ferritin heavy chain, P02794; light chain, P02792; mitochondrial ferritin, Q8N4E7), *S. purpuratus *(Spur1, XP_001184706; Spur2, XP_796152), *D. melanogaster *(Dmel1, NP_524873; Dmel2, AAF07879; Dmel3, NP_572854), *C. elegans *(Cele1, NP_504944; Cele2, NP_491198), *Suberites domuncula *(Sdom1, CAC84556; Sdom2, CAC84555), *Hydra vulgaris *(Hydra, ABC25029), *Arabidopsis thaliana *(Athal, NP_195780), and *Escherichia coli *(Ecoli, NP_416418). Red color indicates 90% consensus and blue color 40% consensus. (B) A Bayesian protein was generated using aligned ferritin sequences including bacterial ferritin from *E. coli *as out-group. This analysis revealed that *Platynereis *ferritin 1 group near to ferritins from the sponge *Suberites domuncula *and the sea urchin *S. purpuratus *whereas the *Platynereis *ferritin 2 isoform groups near to the ferritins from *Hydra *and *Drosophila*. Posterior probabilities are plotted at the nodes. The scale bar represents the substitutions per site according to the model of amino acid evolution applied.

**Figure 5:**
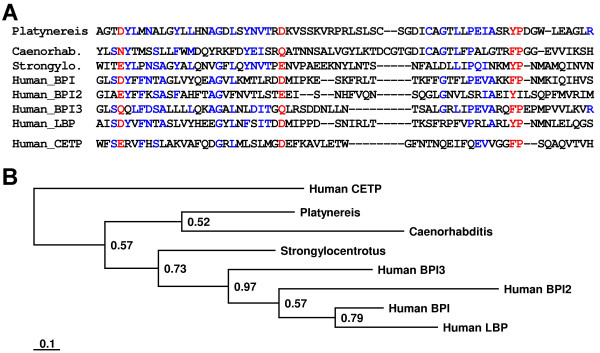
**Sequence alignment of the potential *Platynereis *BPI with homologues from other organisms**. (A) The partial sequence of the BPI from *P. dumerilii *(Platynereis, AM697673), was aligned with sequences from *C. elegans *(Caenorhab., NP_510689), from *S. purpuratus *(Strongylo., XP_001192950), and from *Homo sapiens *(Human_BPI, NP_001716; Human_BPI2, NP_777592; Human_BPI3, NP_079503; Human_LBP, NP_004130) As out-group we used human cholesteryl ester transfer protein (Human_CETP, NP_000069). Red color indicates 80% consensus and blue color 40% consensus. (B) A Bayesian protein tree was generated using the aligned region of proteins and we found that *Platynereis *BPI exhibit highest relation to the *C. elegans *BPI. However, the analysis reveals that BPI from *P. dumerilii *is more ancestral in sequence when compared to BPI from *C. elegans*. Posterior probabilities are plotted at the nodes. The scale bar represents the substitutions per site according to the model of amino acid evolution applied.

**Figure 6:**
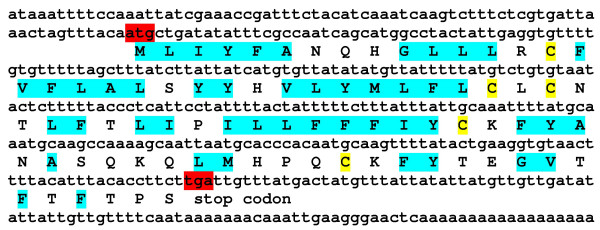
**cDNA sequence and deduced amino acid sequence of a putative antimicrobial peptide from *P. dumerilii***. The identified cDNA of a putative antimicrobial peptide (AM697680) is shown including the deduced protein sequence. Start and stop codons are indicated by red shading, hydrophobic amino acids by gray shading and cysteine residues potentially involved in disulfide bridging by yellow shading.

In addition, the immune-induced expression of following effector molecules were documented in *P. dumerilii*: (I) α-amylases are able to hydrolyze 1,4-alpha-D-glucosidic linkages in oligo- and polysaccharides and belong to a multigene family [30]. Confirming their potential antimicrobial activity human salivary α-amylase has been reported as an effective inhibitor of *Neisseria gonorrhoeae in vitro *[31]. (II) One deduced protein exhibits sequence similarities to the serine protease inhibitors of the migratory locust *Locusta migratoria *[32]. Many serine proteinase inhibitors have evolved in vertebrates and invertebrates to regulate vital serine proteinase cascades including melanisation [33,34], hemostasis, or complement system [35,36]. (III) We identified one protein sharing sequence similarities to a family of immune-induced proteins from insects with not yet elucidated function [15,37]. These proteins contain a reeler domain that is also present in human reelin and stromal cell derived factor receptor 2 homologue. (IV) Ferritin serves as an evolutionarily conserved molecule for the iron-withholding strategy in innate immunity [38] and is strongly induced after immune-challenge in vertebrates and invertebrates [39,40]. Accordingly, we identified transcripts encoding two related ferritin subunits from *Platynereis*. Alignment and phylogenetic analysis of the ferritin isoforms revealed that *P. dumerilii *ferritin 1 groups near to known ferritins from the sponge *Suberites domuncula *and from the sea urchin *S. purpuratus *whereas the second isoform groups near to the ferritins from *Hydra *and *Drosophila *(Fig. 4). The obtained phylogenetic tree needs careful interpretation, but implicates at least that the identified *P. dumerillii *ferritins represent orthologous genes that arose during early animal evolution whereas ferritins from *Caenorhabditis*, *Drosophila *and human are obviously encoded by paralogous genes, and ferritin gene duplications occurred independently in different lineage bifurcations. Interestingly, *Drosophila *ferritins are secreted proteins [38] that exhibit highest deviation in sequence when compared with other analyzed ferritins. In contrast, vertebrate ferritins are localized in the cytoplasm and in mitochondria whereas the plant ferritin is localized in chloroplasts. (V) We found a protein sharing highest sequence similarities with bactericidal permeability-increasing proteins (BPI) from other organisms (Fig. 5). BPI has been recognized to play an important role in innate immunity against Gram-negative bacteria by direct microbicidal and by endotoxin-neutralizing action [41,42]. In humans, several isoforms of BPI are present and form a protein family to which lipopolysaccharide-binding protein (LBP), cholesteryl ester transfer protein (CETP), and phospholipid transfer protein (PLTP) also belong [43]. A phylogentic analysis of the *Platynereis *BPI sequence with counterparts from other animals determined highest similarity to BPI from *C. elegans*. (VI) Several identified transcripts encode hypothetical proteins with no significant blastX match, but two of them exhibit features of antimicrobial peptides [44]. For example, a cationic and a hydrophobic peptide with predicted α-helical structures were identified. The latter has cysteine residues that may form disulfide bridges (Fig. 6).

### Quantitative real-time RT-PCR analyses

**Figure 7:**
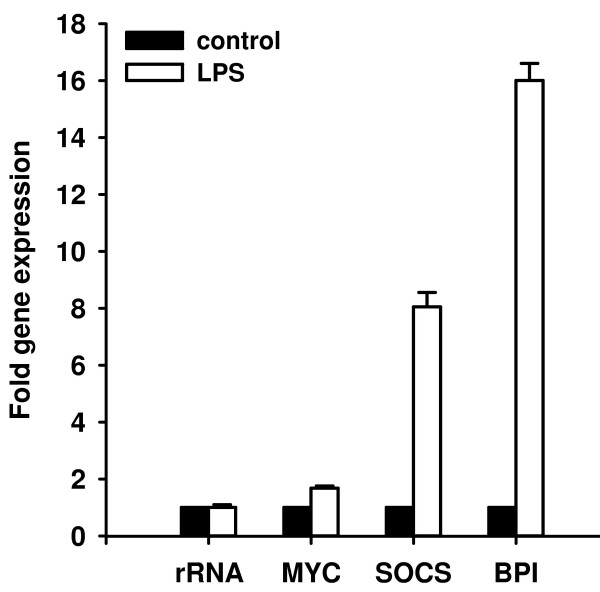
**Quantitative real-time RT-PCR analysis of transcriptional levels of selected *Platynereis *genes in response to immune challenge**. The mRNA levels of selected genes in immune challenged animals (black bars) were determined and are shown relative to their expression levels in untreated animals (white bars). The transcription rates of myc, SOCS, and BPI genes were found to be increased over 1.6, 8, and 16 fold, respectively, in response to LPS injection. In contrast, the amount of 18 S rRNA transcripts were not significantly influenced. Results represent mean values of three independent determinations ± S.D.

In order to precisely quantify gene expression of selected genes, we used real-time RT-PCR and total RNA from LPS-injected and untreated animals (Fig. 7). In *Platynereis*, we found that the transcriptional rates of myc were induced about 1.6 fold, of SOCS about 8 fold, and of BPI over 16 fold in response to immune challenge. In contrast, the expression of the house-keeping gene 18 S rRNA was not influenced by the treatment.

## Conclusion

In order to elucidate immune responses from a lophotrochozoan model organism that has been recognized as a slow-evolving Bilateria, we analyzed the immune-related transcriptome of the marine annelid *P. dumerilii*. Using the suppression subtractive hybridization method we identified 35 genes encoding proteins potentially involved in *Platynereis *immunity. Comparison with the EST database of *P. dumerilii *[45] revealed that 70% of the clusters represent novel genes, although 21,762 EST clones with an average length of 1,277 base pairs were available. This confirms the suitability of the used method and increases the number of identified genes from this polychaete.

The immune-induced expression of myc, SOCS, and BPI was confirmed by quantitative real-time RT-PCR analyses. In addition, phylogenetic analyses implicate that *Platynereis *immunity-related genes are more related to counterparts from Deuterostomia than are those from Ecdysozoa (e.g. *Drosophila *and *Caenorhabditis*). Furthermore, in the case of BPI no protein with sequence similarities is present in the fully sequenced genome from *D. melanogaster*, whereas proteins from humans, sea urchin, and *Caenorhabditis *exhibit significant sequence similarities to the identified homologue from *Platynereis*. Taken together, these findings are in line with recent reports that validate that *P. dumerilii *and human genes are evolutionarily more primary in structure and sequence than are those of fast-evolving animals where these initially complex genes have been secondarily simplified in some cases [8,9]. In conclusion, our results regarding the immune-induced genes from *Platynereis *will be a valuable resource for subsequent polychaete specific immune studies in particular and comparative lophotrochozoan/bilaterian immune studies in general.

## Methods

### Immune challenge of adult *Platynereis dumerilii *and RNA isolation

Adult *Platynereis dumerilii *(strain Berlin) were obtained from H.-D. Pfannenstiel, Institute of Biology, Free University of Berlin, Germany. 10 μl sample volume, corresponding to 1 μg LPS (purified *Escherichia coli *endotoxin 0111:B4, Cat. No.: L2630, Sigma, Taufkirchen, Germany) per animal were injected dorsolaterally into the coelom using 1 ml disposable syringes and 0.4 × 20 mm needles mounted on a microapplicator. 8 h post injection animals were homogenized in liquid N_2 _and total RNA was extracted using the Qiagen RNeasy kit according to the instructions of the manufacturers. RNA integrity was confirmed by ethidium bromide gel staining and quantities were determined spectrophotometrically [46].

### Construction of a subtracted cDNA library using the SSH method

In order to identify differentially expressed genes during immune response the SSH method was performed using RNAs from immune-challenged and control animals, the SMART PCR cDNA synthesis Kit (Clontech, Mountain View, CA, USA), and the PCR-Select cDNA subtraction Kit (Clontech), according to the protocols of the manufacturer.

### Colony PCR and blot hybridization

Colony PCR was performed on 288 randomly picked colonies with vector specific primers T7-promotor: 5'-TAATACGACTCACTATAGGG-3' and SP6: 5'-ATTTAGGTGACACTATAG-3' (purchased from Thermo electron, Waltham, MA, USA) using a Biometra PCR cycler and the Red Taq PCR system (Sigma, Taufkirchen, Germany). Used PCR conditions were: denaturation at 95°C for 3 min followed by 30 cycles of denaturation at 95°C for 15 s, annealing at 43°C for 15 s, and extension at 72°C for 60 s. A final 7-min 72°C step was added to allow complete extension of the products. 1 μl of resulting PCR products were identically spotted onto two sheets of positively charged nylon membranes (Roche, Lewes, United Kingdom). Membranes were dried and UV cross-linked using a BioRad UV cross-linker (BioRad, München, Germany), according to the instructions of the manufacturer. Digoxigenin labeled probes for hybridization were generated using secondary PCR products of subtracted and non-subtracted cDNAs and the Dig-High Prime Labelling kit (Roche, Lewes, United Kingdom). Hybridization, washing, and detection of digoxigenin labeled DNA was performed in accordance to the user guide instructions of the Dig Easy Hyb Granules, Dig-Wash and Block Buffer Set, Anti-Digoxigenin-AP and NBT/BCIP ready-to-use tablets (Roche, Lewes, United Kingdom).

### Sequencing and computer analysis of cDNA sequence data

Plasmid isolation of 140 positively screened colonies was performed with the Fast MiniPrep kit (Eppendorf, Hamburg, Germany) and purified plasmids were custom sequenced by Macrogen Inc. (Seoul, South-Korea). Sequences were used to identify similar sequences of SWISS-PROT protein sequence database (available at the National Center for Biotechnology Information) using BLASTX program (BLASTX 2.2.13) [47]. InterProScan [48] was used for an integrated search in PROSITE, Pfam, and PRINTS databases at EMBL-European Bioinformatics Institute and to predict signal sequences and transmembrane regions.

### Quantitative real-time PCR

Quantitative PCR was performed with the real-time PCR system Mx3000P (Stratagene, La Jolla, California, USA) using the FullVelocity SYBR^® ^Green QRT-PCR Master Mix (Stratagene), according to the protocols of the manufacturer. In order to confirm the subtraction efficiency of constructed cDNA library 1 ng of unsubtracted and subtracted cDNA, respectively, was used to amplify 18S rRNA, α-tubulin, BPI, and SOCS. Used primers were: 18S rRNA-forward: 5'-ATGGTTGCAAAGCTGAAACT-3', 18 S rRNA-reverse: 5'-TCCCGTGTTGAGTCAAATTA-3', the universal primers α-tubulin-forward: 5'-GCCAACCAGATGGTCAA-3' and α-tubulin-reverse: 5'-GCTTGGTCTTGATGGTG-3', BPI-forward: 5'-TTCAAGCAAGGTCCGTCCAA-3', BPI-reverse: 5'-CCTGCTTCCAGCCATCCATC-3', SOCS-forward: 5'-CAGATGGCCTGCAGGTACCAA-3', and SOCS-reverse: 5'-TCGAGTCAATGCCACTGTCCA-3'. For gene expression analyses, we used 100 ng total RNA per well and primers described above. In addition, primers MYC-forward: 5'-GCCCCTGCAGTTTCAGTTTCA-3' and MYC-reverse: 5'-TGGGGAGACAGGTTCGGATTT-3' were used to amplify myc transcripts.

### Sequence alignments and phylogenetic analyses

Sequence alignments were computed using the blosum62 algorithm [49,50]. For phylogenetic reconstruction, we used the software package MrBayes 3.1.2 [51], which combines Bayesian inference and Markov chain Monte Carlo convergence acceleration techniques known as Metropolis coupling. The best fixed-rate model of amino acid evolution was determined by model jumping among nine possible models. The model with the overall highest posterior probability was Jones model [52] for the myc protein after 5 × 10^6 ^generations and blosum62 model [53] for BPI after 10^6 ^generations and also for ferritins after 10^6 ^generations. We used convergence diagnostic (i.e., the standard deviation of split frequencies) to determine whether the run length is sufficient. The average standard deviation of split frequencies was 0.0021 for BPI, 0.0049 for the ferritins, and 0.00024 for the myc, respectively. This indicated that the two chains that were run converged on similar results in all cases. The 50% majority rule tree presented here was constructed from all sampled trees with the first 25% of all trees ignored as burn in. Trees were visualized with TREEVIEW 1.6.6 [54]. Posterior probabilities plotted at the nodes can be interpreted as the probability that the tree or clade is correct [55].

## Competing interests

The author(s) declare that they have no competing interests.

## Authors' contributions

BA designed and carried out the experiments, performed the analyses, and drafted parts of the manuscript. AV injected the animals, participated in its design and coordination, and drafted parts of the manuscript. All authors read and approved the final manuscript.
